# Research impact in the community-based health sciences: an analysis of 162 case studies from the 2014 UK Research Excellence Framework

**DOI:** 10.1186/s12916-015-0467-4

**Published:** 2015-09-21

**Authors:** Trisha Greenhalgh, Nick Fahy

**Affiliations:** Nuffield Department of Primary Care Health Sciences, University of Oxford, New Radcliffe House, Radcliffe Observatory Quarter, Woodstock Road, Oxford, OX2 6GG UK

**Keywords:** Primary care, Public health, Research impact, Knowledge translation

## Abstract

**Background:**

The 2014 UK Research Excellence Framework (REF2014) generated a unique database of impact case studies, each describing a body of research and impact beyond academia. We sought to explore the nature and mechanism of impact in a sample of these.

**Methods:**

The study design was manual content analysis of a large sample of impact case studies (producing mainly quantitative data), plus in-depth interpretive analysis of a smaller sub-sample (for qualitative detail), thereby generating both breadth and depth. For all 162 impact case studies submitted to sub-panel A2 in REF2014, we extracted data on study design(s), stated impacts and audiences, mechanisms of impact, and efforts to achieve impact. We analysed four case studies (selected as exemplars of the range of approaches to impact) in depth, including contacting the authors for their narratives of impact efforts.

**Results:**

Most impact case studies described quantitative research (most commonly, trials) and depicted a direct, linear link between research and impact. Research was said to have influenced a guideline in 122 case studies, changed policy in 88, changed practice in 84, improved morbidity in 44 and reduced mortality in 25. Qualitative and participatory research designs were rare, and only one case study described a co-production model of impact. Eighty-two case studies described strong and ongoing linkages with policymakers, but only 38 described targeted knowledge translation activities. In 40 case studies, no active efforts to achieve impact were described. Models of good implementation practice were characterised by an ethical commitment by researchers, strong institutional support and a proactive, interdisciplinary approach to impact activities.

**Conclusion:**

REF2014 both inspired and documented significant efforts by UK researchers to achieve impact. But in contrast with the published evidence on research impact (which depicts much as occurring indirectly through non-linear mechanisms), this sub-panel seems to have captured mainly direct and relatively short-term impacts one step removed from patient outcomes. Limited impacts on morbidity and mortality, and researchers’ relatively low emphasis on the processes and interactions through which indirect impacts may occur, are concerns. These findings have implications for multi-stakeholder research collaborations such as UK National Institute for Health Research Collaborations for Leadership in Applied Health Research and Care, which are built on non-linear models of impact.

## Background

The 2014 UK Research Excellence Framework (REF2014) was the first national exercise to measure the impact of research in the higher education sector (Box 1) [1]. It reflected a growing policy interest in demonstrating the benefits of investment in academic research and reducing the waste that occurs when findings are not implemented [2, 3].

The literature on research impact has been summarised in several recent reviews [4–7]. Researchers often assume a direct and linear link between a study and subsequent impact, achieved through academic publications or corresponding lay summaries [8]. Policymakers may assume that they can commission targeted research to solve policy problems. In reality, these ‘knowledge-driven’ and ‘problem-solving’ mechanisms of impact are uncommon [9]. Clinicians rarely read published research or consciously follow guidelines [10]; and policymakers ask different questions and operate to very different logics, timescales and value systems from researchers [11, 12].

That is not to say that a direct and linear link between research and impact never occurs. If a research finding is simple, unambiguous and uncontested; if it aligns with people’s values and predictions (especially those of local opinion leaders) and/or with a policy window; if its implementation can be trialled on a small scale before practitioners commit; if mechanisms exist (or can easily be made to exist) to provide timely reminders and feedback on the change; if implementing the finding saves money or becomes a legal or professional requirement; and if the implementation is generously resourced and incentivised, the research finding may be taken up directly [12–14].

More commonly, impact from research occurs indirectly. Among clinicians, this happens via ‘mindlines’ (that is, collectively generated and socially shared tacit knowledge, developed in professional communities of practice) [10]. In the policy setting, it occurs when researchers and policymakers, through repeated interaction over time, come to better understand each other’s worlds and develop goals that are compatible if not fully aligned. This process has been described variously as ‘percolation’ [15], ‘linkage’ [16] and ‘pragmatic muddling through’ [17]. In the commercial sector, it occurs through two-way secondments and ‘value co-creation’ by the entrepreneurial university [18–20]. The principle of co-creation—the collaborative generation of knowledge in its context of application by academics working with other partners, sometimes referred to as ‘Mode 2 knowledge production’ [21]—also underpins networked models of research such as the UK’s Collaborations for Leadership in Applied Health Research and Care (CLAHRCs), in which universities and local National Health Service organisations collaborate to identify research priorities, undertake applied research and build research capacity in the service sector [22].

Contemporary health research systems are made up of multiple stakeholders with competing interests, and are characterised by (more or less) productive conflicts and bidirectional knowledge exchange. They illustrate why the unenhanced ‘logic model’ of impact, comprising inputs (research funding) → activities (research) → outputs (e.g. papers, guidelines) → outcomes (e.g. changed clinician behaviour, new service models) → impacts (e.g. reduced mortality), is increasingly viewed as over simplistic [4, 5, 18]. Some scholars of impact (notably Buxton and Hanney, who developed the widely used payback framework [23]) have proposed making the logic model more ‘permeable’, for example by emphasising the interactions and feedback loops that link researchers, research commissioners, knowledge intermediaries and end-users throughout the research cycle.

Other scholars prefer to depict the research endeavour and its various stakeholders as a complex (adaptive) system [22, 24, 25]. Especially when research findings are complex and ambiguous (e.g. where they illuminate the complexity of a phenomenon rather than providing a definitive answer to a simple question); where they fit poorly with prevailing policy priorities; or where their potential stakeholders are many but agreement between those stakeholders is low (perhaps because of conflicts of interest), logic models lose their predictive power [8, 12, 16]. In such cases, it is particularly important to study the processes and interactions by which research priorities are set, studies planned and executed, data analysed, findings made public and implications debated [12, 26, 27]. If we overlook these interactions, there is a risk that any impact assessment exercise will be reductive and naïve.

REF2014 produced a unique and important database of impact case studies, each describing a body of research and impact beyond academia (Box 1). Some researchers have begun to use computerised text mining techniques to generate ‘big data’ from all 6,975 impact case studies in this dataset (save for a tiny fraction of commercially or otherwise sensitive case studies that were redacted) [28]. In this study, we sought to complement that work by manually reading and coding a smaller (though still substantial) sample of case studies submitted to a single sub-panel (A2: Public Health, Health Services Research and Primary Care). Our research questions were (1) What kinds of research designs and impacts were described in these submissions?; (2) What models and mechanisms were invoked to account for the impacts?; and (3) To what extent did the format and scoring system support the assessment of direct, indirect and co-produced impact?

## Methods

Our study consisted of two elements: a descriptive survey of all 162 impact case studies submitted to sub-panel A2 in REF2014, and a more in-depth analysis of four of these case studies selected for maximum variety in study design, range of impacts and mechanism of impact; all were considered to illustrate some aspect of good practice. Queen Mary University of London Research Ethics Committee approved the study in December 2014 (QMERC1388b). Informed consent was not required because all documents were in the public domain.

TG read and re-read a sub-sample of 30 impact case studies and used these inductively to develop a draft data extraction framework on a Microsoft Excel spreadsheet, informed by a systematic literature review undertaken in parallel [4]. She then went through the full set of 162 cases and extracted data on study design(s), impacts, assumed mechanism of impact (coded as direct, indirect, or co-produced or ‘Mode 2’), and active efforts described by the research team to achieve impact. NF independently coded a random 20 % sample of the case studies and checked TG’s interpretations; differences were resolved by discussion.

Judgement was needed to code and categorise free-text descriptions, especially in the last category. For example, many case studies mentioned one-off coverage of the research findings by the lay press, but this alone was considered insufficient to count as proactive engagement with the media. Similarly, linkage with policymakers or front-line clinicians was counted as ‘active efforts’ if there was evidence that these links were strong and ongoing, preferably pre-dating the research and continuing through the research period and beyond it. If the research had been commissioned (e.g. by policymakers, industry or other interest groups), this ‘pull’ was also classified as a form of linkage. The 31 impact templates (which described the infrastructure for achieving impact in submitting institutions) were also coded.

For the in-depth analysis, we selected a maximum variety sample to illustrate the full breadth of research designs and approaches to impact, and within each design, selected an example ‘model of good practice’ (that is, a study whose impact had been very substantial and also evident across different categories of impact). For each of the four included case studies, we conducted an interpretive analysis considering how the research was linked to the claimed impact, taking account of the different framings and mechanisms of impact described in the introduction. In these four case studies, we also read the papers from the underpinning research and contacted the authors by email and telephone for their own account of the case. Our initial sample was five such studies but the lead author of one responded (positively) only to our initial email and ignored subsequent contact, so after checking to ensure that no further themes had been identified thus far in the excluded case, we reduced our sample to four.

## Results

As predicted by RAND Europe [7], the case study format generally contained rich information, allowing us to make a reasonably confident judgement about the nature and quality of the research, the depth and quality of interactions, and the nature and mechanism of claimed impact. All 162 case studies are in the public domain [29]; the detailed dataset extracted from them is available from the authors; our inter-rater reliability in coding was 89 %; most discrepancies were minor and stemmed from ambiguities in the coding framework.

**Fig. 1:**
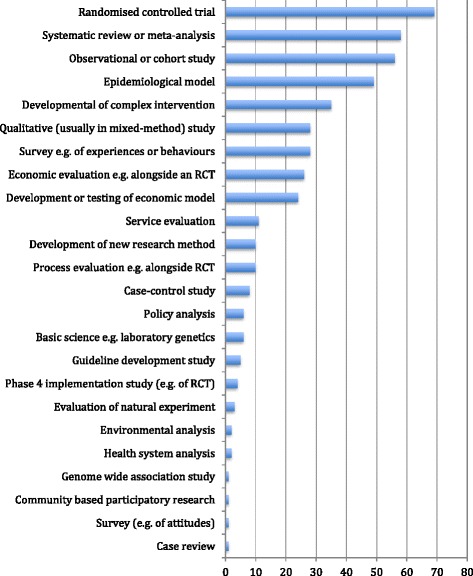
Study designs used in 162 impact case studies submitted to sub-panel A2 of REF2014. *RCT* randomised controlled trial

Figure 1 shows the research designs represented. Most case studies included more than one design (mean 2.7). Quantitative methods predominated, especially randomised controlled trials, systematic reviews with statistical meta-analysis, longitudinal cohort studies and modelling studies (epidemiological or economic). Twenty-eight (around one in six) described some qualitative research, but in all but two, the qualitative research was presented as preliminary (hypothesis-generating) or as a minor element of a mixed-methods study (typically, attitudes of participants in a randomised trial).

Most case studies identified more than one target audience for the research findings: policymakers in 133 (including the National Institute for Health and Clinical Excellence, which leads most national guideline development in the UK), clinicians in 88 and industry in 15. Few (8 our of 162) explicitly identified patients and/or the lay public as an audience, which was especially surprising given that every submitting institution claimed in its impact template that ‘patients and the public’ were a key audience for its work.

In 161 of the 162 case studies, the main mode of impact depicted was direct; in nine of these there was also considered to be an element of co-production and in two, the authors proposed an element of indirect (‘enlightenment’) impact. In one case study, impact was depicted as mainly co-produced with a lesser component of direct impact. Some case studies talked in general (and sometimes unconvincing) terms about promoting public debate.

**Fig. 2:**
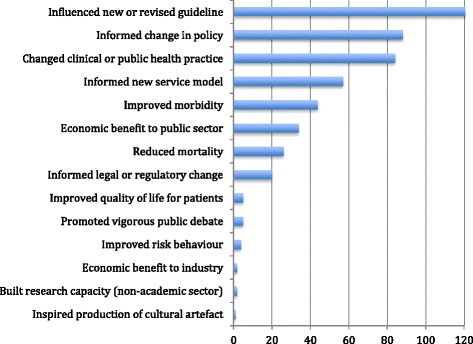
Main impacts described in 162 case studies

The main impacts described are shown in Fig. 2.

Each case study claimed impact in (on average) three different areas. In more than two-thirds (122/162), research had influenced a clinical guideline and in more than half, it had had some influence on an international, national or local policy (88/162) and/or changed clinical or public health practice (84/162). Less commonly, there was evidence of a new service model (57/162), or improvement in morbidity (e.g. disease progression, symptom severity; 44/162), mortality (i.e. clear evidence of improved survival; 25/162) or quality of life (5/162) attributable to the research. In 32 case studies, research was linked to cost saving to the health service and in three to documented profits for industry.

**Fig. 3:**
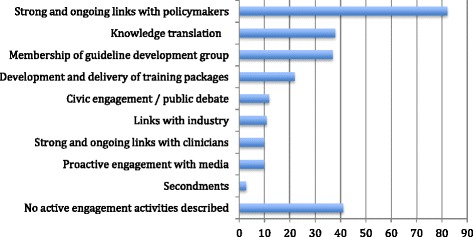
Active efforts by researchers to achieve impact from their research

Active efforts described by research teams to maximise impact are summarised in Fig. 3. In 40 of the 162 case studies, researchers described no active efforts to achieve impact. This is perhaps not surprising because the official REF guidance did not specifically advise them to include such descriptions. Encouragingly, in over half the case studies (82/162), and notably in those that described commissioned research for the Department of Health (England) or Chief Scientist’s Office (Scotland), there was evidence of strong and ongoing links with policymakers. It was also common for submitting researchers to be represented on (and perhaps be invited to chair) guideline development groups.

Much less commonly (in only 10/162 case studies) was there evidence of strong linkage with front-line clinicians, though again such links may have been assumed and gone undescribed. Surprisingly, only a minority of case studies (38/162) explicitly described efforts to ‘translate’ their research findings into formats accessible to policymakers, front-line clinicians or the lay public (for example by producing leaflets or online materials designed for the needs and learning styles of designated audiences), and only 22 of 162 described a specific training package developed for, and delivered to, a particular non-academic audience. There also appeared to be limited engagement with industry, but this may have been due to the fact that most industry-relevant studies were returned to other sub-panels.

The four case studies analysed in depth—‘Bell’s Palsy’ from Dundee, ‘Sudden Infant Death Syndrome’ from Bristol, ‘The Impact of Social Inequality’ from York, and ‘Developing the Evidence Base on Lay Health’ from Leeds Beckett—are downloadable from the Higher Education Funding Council for England website [29] and their key features are summarised in Table 1. They illustrate aspects of good practice and/or examples of the range of approaches to achieving impact.

**Table 1:** Four in-depth impact case studies: summary of key features

Short title	Study design	Main impacts	Main mechanisms of impact
BELLS (Dundee) [36]	Randomised controlled trial	Improved cure rate	Commissioned as ‘high clinical priority’ study by Health Technology Assessment Programme
		Reduced referral to hospitals	Ex ante and ongoing engagement of clinicians
			Widespread involvement of clinical research networks nationally
			High-impact publication in international journal
Cot death (Bristol) [37]	Case–control	Reduced mortality	Well-established and mature programme of ‘niche’ research
			Ex ante and ongoing engagement of third sector charity
			Skilled knowledge translation (working with knowledge translation experts) to disseminate key messages for lay audiences
			Commitment of researchers to the ‘moral work’ of linking the contribution of research participants (bereaved parents) and potential beneficiaries (new and prospective parents)
Social inequality (York) [38]	Systematic review of observational studies	Shifting the focus of public debate	Energetic and proactive dissemination campaign run through a newly established charitable trust
		Achieving political commitment to addressing the issues	Extensive lobbying of prospective and existing politicians and policymakers
		Authors’ input to commissions and working parties	Primary focus on outputs for a lay/civic audience with ‘academic’ outputs as a secondary priority
		Production of cultural artefacts	Researcher commitment to ‘moral work’
Lay people in public health (Leeds Beckett) [39]	Co-production, systematic review, service evaluation	Lay health trainer programmes established locally	Local multi-stakeholder partnerships
		Online public information resource	Co-production model
			Emphasis on ensuring all voices were heard
			Ex ante linkage with (and preferred provider status to) national policymakers

The Bell’s Palsy case study from the University of Dundee (Appendix 1) described a well-conducted clinical trial in a community setting whose findings were important, definitive and easy to understand. The study’s impact was direct and readily captured by existing data systems. It was aided by the high quality of the research, the commissioned mode of research (hence, a ‘policy pull’) and extensive ex ante links with front-line general practitioners and the emerging networks for general practice research in Scotland.

The case study from the University of Bristol on sudden infant death syndrome (Appendix 2) illustrates the rare but effective approach of developing proactive links with a ‘niche’ patient charity and paying careful attention to high-quality knowledge translation for a lay audience. It also shows how researchers’ personal, emotionally engaged, ethical commitment to reducing harm through research can provide impetus and justification for the time spent on impact activities.

Passionate commitment to ethical implications was also a feature of Wilkinson and Pickett’s study of income inequality from the University of York (Appendix 3). Strikingly—and very unusually in our sample of 162—the societal impact of this research (promoting debate, influencing political and policy decisions in the UK and internationally) seemed to have been the authors’ primary objective, while publication in ‘high impact’ journals appeared secondary.

Our last case study, Developing the Evidence Base on Lay Health (Appendix 4) was selected because it was the only one in this sample of 162 that was based primarily on co-produced (Mode 2) research. Raw scores for individual impact case studies were not published, but the overall impact submission (template plus three case studies) from Leeds Beckett did not score well. The prescribed format of the impact case study (describe the research and then describe the impact) may have made it difficult for the authors to emphasise the strengths of this work. As the account in Appendix 4 illustrates, the authors did not depict a linear and causal link between ‘upstream’ research and ‘downstream’ impact (this was an impossibility inherent to the Mode 2 design). The authors’ emphasis on principles (inclusivity, inter-sectoral partnerships) and activities (relationship-building, ongoing dialogue with policymakers) may have been unfavourably compared with ‘hard’ metrics of impact in other case studies that had been narrativised in logic model frameworks.

## Discussion

This detailed review of the impact case studies submitted to a single sub-panel in REF2014 showed that the underpinning research for cases submitted to this sub-panel was overwhelmingly university-led and consisted mainly of clinical trials, meta-analyses and modelling studies. Impacts were mostly in the form of changes to guidelines and policies, and the main mechanism of impact was depicted as direct knowledge-into-practice, often achieved via ex ante linkage with policymakers and representation on guideline development groups. Few case studies described patient-relevant changes in morbidity, mortality or quality of life or documented whether the evidence-based guideline was actually being followed. Only a handful placed significant emphasis on indirect impact or collaborative knowledge production. There was a striking mismatch between institutions’ claims to have engaged with ‘patients and the public’ (universally made in impact templates) and the limited range and depth of activities oriented to achieving this described in the individual case studies.

This study illustrates how detailed manual analysis of a small sample of case studies can complement the more automated ongoing analysis of the wider REF dataset. Text mining of the REF dataset has already produced useful quantitative data (such as frequency statistics on different countries to which impact had spread) [28]. There is, however, a trade-off between breadth and depth, given that automated analysis of a massive dataset is unable to tease out meaningful narratives or detailed mechanisms. Further research could fruitfully explore where and how in-depth qualitative analysis could inform and complement text mining approaches and vice versa.

The structure of the impact case study in REF2014 (a four-page document divided into ‘summary’, ‘underpinning research’ and ‘impact’ along with space for references and corroborating sources) arguably implied a direct and linear link between a programme of research and its subsequent impact. Perhaps because of this, and also because of the context of REF2014 (UK higher education’s opportunity to demonstrate that it was internationally competitive and value for money), almost all impact case studies in our sample were presented using a linear, ‘logic model’ framing.

To some extent, this framing worked in that it allowed research teams to draw out the link between particular research studies and resulting changes in clinical guidelines, health policies (international, national or local) and, to a lesser extent, health outcomes and/or some form of cost saving. The narrative form also allowed research teams to express their passion for the topic, explain why it mattered and convey how their research did ‘moral work’ (reducing harm and/or achieving social justice). But as others have predicted previously (see Background), the implicit logic model framing seemed to both invite and reward ‘hard’, quantitative experimental studies and computational models that had clear, measurable and attributable short-term impacts (most commonly, incorporation into guidelines).

Whilst one interpretation of our data is that impact *is* largely linear and best achieved through quantitative empirical studies (hence, such study designs are ‘stronger’), another is that the more diffuse impacts from fields such as social science and policy research could not be captured, so institutions made a strategic decision not to submit them as impact case studies. The design of this study allows no conclusions about research whose impacts were not submitted to REF2014. But (given that sub-panel A2 potentially covered a very broad church of research designs relevant to primary care and public health) the dearth of designs grounded in the social sciences (such as qualitative studies, evaluations of natural experiments and policy analyses) in impact submissions is consistent with previous claims that such work rarely produces direct and readily measurable impacts [8]. The format of the REF impact case study (strong emphasis on measurable impacts that could be tracked back to the study reported in the ‘research’ section) allowed direct but not indirect flows of influence to be demonstrated. This may have created a bias towards biomedical and epidemiological research and away from health services and policy research (in which impact is inherently less linear, more complex and multi-stakeholder, and thus harder to demonstrate).

None of the case studies in this sample framed impact in terms of the productive (and organic and reciprocal) interactions among organisations and sectors described by impact scholars who have taken a ‘complex systems’ perspective [8, 21, 22, 24–27]. Indeed, perhaps our most significant empirical finding is the mismatch between the sophistication of theoretical approaches to assessing impact published in the specialist ‘research on research’ literature (see ‘Background’) and the direct and linear way in which the research-impact link was actually depicted and scored in REF2014 submissions.

Almost entirely absent from this sample of impact case studies was any acknowledgement of power differentials or conflicts of interest in the research-impact link. Few case studies describing co-produced research were submitted, but as the example in Appendix 4 illustrates, even those that were submitted did not highlight clearly how power was shared during the research or the extent to which power was redistributed as a result of that research (so-called emancipatory benefits [30]). Again, this may have been because the REF template did not ask for such information and its linear format implicitly discouraged discussion of such matters. Yet the emerging evidence from CLAHRCs suggests that issues of power and conflict loom large in co-production models of research, and that any impacts are likely to depend heavily on how these issues are handled [22, 31].

Our findings thus suggest that that the high impact scores for Medicine in REF2014, whilst commendable up to a point, are no cause for complacency. The final report from REF Main Panel A strongly upheld the usefulness of the case study format and cautioned against reducing any future exercises to automated metrics [32]. But that conclusion does not mean that the outline structure used in REF2014 is optimal. In particular, our data suggest (though they fall short of proving) that those whose research had indirect, long-term and (therefore) more contestable impacts and those involved in participatory (co-production) models may have been discouraged from submitting impact case studies in this exercise. We know of no comparable exercises in other countries that could help answer the important question: might a different structure for reporting impact support the submission of a broader range of research?

On the basis of our findings, and with a view to ensuring that future research assessment exercises do not inadvertently create a perverse incentive to focus on ‘safe’ impacts at the expense of wider engagement activities, we suggest some changes to the design of the impact case study. First, systematic reporting of the processes and activities oriented to achieving impact should be a requirement. Indeed, in studies such as health policy research where impact is largely indirect, these processes and activities should be allocated a substantial proportion of the overall score. Research teams should not be penalised for impact activities (such as building relationships with policymakers, professional bodies or citizens) that are worthwhile in the long term but unlikely to map to specific, measurable impact metrics in the timescale of the assessment.

Second, we suggest that impact case studies from Mode 2 research collaborations such as action research partnerships (Appendix 4) or CLAHRCs should be assessed differently from more conventional study designs such as randomised trials. In particular, assessment should recognise that in such designs, ‘research’ and ‘impact’ are not always separate and sequential stages but may be two dimensions of co-produced activity; and that activities oriented to effective governance may be crucial to the success of this activity.

Third, given the importance shown in this study of individual researcher enthusiasm and commitment in achieving research impact, it may be worth reconsidering the current model of assigning the entire impact score to the researcher’s current institution. If researchers move institutions, they are likely to continue their efforts to follow through on past research undertaken elsewhere. And such work would be in the public interest—but it will not bring benefit to the new institution.

## Conclusion

Our findings highlight both the strengths and limitations of the prevailing assessment system and raise questions about impact measurement more generally. REF2014 both inspired and documented significant efforts by UK researchers to achieve impact. But whilst these efforts, and the short-term direct impacts described in most impact case studies, should be celebrated, further thought needs to be given to how indirect and longer-term impacts can be fully and fairly captured.

## Figures and Tables

**Fig. 4:**
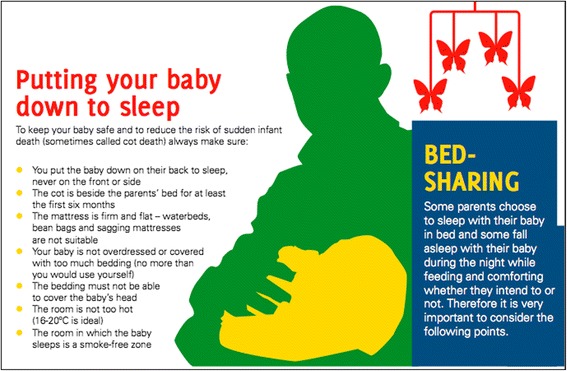
Page from UNICEF leaflet designed as knowledge translation from the Bristol cot death research. (Reproduced with permission from UNICEF Baby Friendly Initiative)

## Appendix 1: A commissioned randomised controlled trial

‘BELLS’ was a large randomised trial conducted in general practices across Scotland, commissioned by the Department of Health via its Health Technology Assessment (HTA) Programme. The target condition, Bell’s Palsy, was sufficiently common that most general practitioners (GPs) would see a case every year or two, and there was a high level of clinical uncertainty about the best treatment. The trial was simple and easily implemented: each newly presenting patient received an antiviral, a steroid, both, or neither, with placebo pills in double-blind fashion. After a pilot study in two General Practice [regional] Research Networks to confirm feasibility, the trial was rolled out to over half of all general practices in Scotland—immediately achieving high clinician engagement for subsequent dissemination of findings. The primary outcome (full recovery at 3 and 9 months) was meaningful to both doctors and patients.

The findings of the BELLS trial were definitive (steroids were better than antivirals), and viewed as very important by clinicians. The principal investigator commented: “[BELLS was] the only study [I have done] where I have had clinicians phoning me to ask about the results in the embargo period when they had a patient in the consulting room” (Professor Frank Sullivan, personal communication, February 2015).

The trial was published in a leading US journal and won a major research prize. This paper and the (longer) HTA report were widely disseminated, actively aided by the HTA who had commissioned the study, and were used to inform national and international guidelines and decision support tools. One mechanism for this was active involvement of the trial’s authors in updating the relevant Cochrane review (the accepted international ‘gold standard’ evidence summary).

Another mechanism was Scotland’s strong General Practice Research Networks, which helped raise awareness among Scottish GPs who had not been part of the trial. Prescribing practice changed quickly as a result. At the time, Scottish general practice had advanced electronic record systems that allowed prescribing patterns for Bell’s Palsy to be tracked using automated data linkage (between the coded diagnosis ‘Bell’s Palsy’ and the coded treatment ‘prednisolone’), so a substantial fall in antiviral prescribing was readily documented.

## Appendix 2: A case–control study of the causes of cot death

The University of Bristol achieved the maximum 4* score for its impact submission in UoA2. One of its case studies, on sudden infant death syndrome (SIDS), described a case–control study that had demonstrated, sequentially over several years, the key risk factors for SIDS—including increased risk from prone and side sleeping positions, bed-sharing, infant covered by bedding, and parental smoking; and the protective effect of breastfeeding. Impacts included change in the legal requirements for investigating cot deaths, widespread changes to public health policy and practice, and a steady and significant reduction in the annual rate of SIDS (partly but not wholly attributable to this research, given that other research teams were also contributing to the evidence base, and social changes such as reduction in smoking prevalence were occurring on the same timescale).

This case study described an unusually high level of activity by the research team to help maximise impact. There was, for example, extensive knowledge translation activity, working closely with UNICEF UK to produce a leaflet ‘Caring for your baby at night’ which encourages breastfeeding and outlines the circumstances in which bed-sharing may be unsafe (Fig. 4). The authors also produced an evidence-based guideline for health professionals to accompany the leaflet. There was active outreach to patient charities (two of the lead investigators were elected, successively, as Vice Chair of the International Society for the Study and Prevention of Perinatal and Infant Deaths, which delivers risk reduction messages in different formats for different countries).

As in the BELLS example, the principal investigators highlighted the importance of working actively and through personal commitment to communicate (initially) the rationale for the study and (subsequently) its findings through a range of media and professional channels.

“The most important part of the mechanism was continuity – we took our own research and pushed its implementation at every stage – we did not leave any part of the implementation to others – having collected and analysed the data, we were in the best position to drive implementation and really understood what could be achieved – probably much more than those who had not been involved in the original studies.” (Professor Peter Fleming, personal communication, February 2015).

Professor Fleming also stressed the ethical duty that he and his team felt to disseminate the findings of the study, which had involved sensitive research on parents who had lost a child to cot death. “Information freely provided by families – many of whom were very deprived – has been used to help other families and prevent babies from dying. I feel that our role was to be a conduit ensuring the information was interpreted and used appropriately”.

## Appendix 3: Evidence synthesis on the link between income inequality and ill health

Epidemiologists Richard Wilkinson and Kate Pickett (University of York) wrote a book, *The Spirit Level*, which synthesised evidence from primary studies that the higher the level of income across a wide range of indicators (“life expectancy, infant mortality, mental health, levels of violence, teenage birth rates, drug abuse, child wellbeing, obesity rates, levels of trust, the educational performance of school children, imprisonment and social mobility”), the worse the society’s physical, mental and social health.

The research demonstrated that the link between inequality and poor health and social outcomes was largely psychosocial and attributable mainly to three factors: low social status, weak social support, and poor quality of early childhood experience.

*The Spirit Level* sold hundreds of thousands of copies worldwide. One of the main impacts (unusually for case studies in our sample) was a significant shift in the focus of public debate. In the light of consistent evidence from multiple studies in numerous contrasting settings, it was much harder to dismiss concerns about the widening gap between rich and poor as ‘the politics of envy’. The conclusion (politically sensitive but strongly supported by the data) was that improvements in social environment, social relations and material environment are likely to improve the psychosocial wellbeing and social functioning of whole societies.

Dissemination strategies were bold and proactive. The authors obtained funding from the Joseph Rowntree Charitable Trust to establish an independent charitable trust whose sole remit was to spread key messages and lobby for change. The Equality Trust [33] produces ‘research digests’ and social media feeds, and coordinates and supports local pressure groups.

Between 2009 and 2013, the authors gave over 600 lectures, workshops and seminars, mostly to lay audiences but also to “government ministries and agencies, the UK Cabinet Office, health authorities, political party conferences, universities, trade unions, faith groups, NGOs, think tanks and charities”. They approached parliamentary candidates in the 2010 UK general election and asked them to “actively support the case for policies designed to narrow the gap between rich and poor”; soon after, 75 signatories (in all main parties) were elected to parliament. The Prime Minister personally endorsed the work and cited the book in speeches. The authors were invited onto working groups that established Fairness Commissions (in various localities) and the national Independent Living Wage Commission.

Dissemination materials for young people were imaginative and diverse. They included a ‘Theatre in Education’ learning module, a novel (Kuan’s Wonderland) and linked study guide, a statistics learning module, a game for learning about economics and inequality, plus collections of videos and other cultural artefacts (see [34]).

One of the authors commented, “I’m … proud of the fact that we managed to create a readable account for people with no expertise or confidence in statistics or reading charts, and that they were empowered to get to grips with important ideas and debates” (Prof Pickett, personal communication, May 2015). Yet the original funding sources were very conventional: an NIHR Career Scientist award and an ESRC grant to produce educational materials for schools.

## Appendix 4: Co-production of research on lay involvement in public health

Researchers from Leeds Beckett University described a co-production (action research) approach to developing lay roles in public health.

The research section of their impact case study lists a number of activities and outputs:

- Three local community-based service development projects (‘health trainers’, ‘community apprentices’ and ‘social prescribing’)
- Three NIHR-funded national ‘public hearings’ of the views of lay health workers, in which the research team played a supportive and evaluative role and also produced an academic paper
- An NIHR-funded literature review, ‘People in Public Health’, of lay roles in public health (though surprisingly, the main publication from this was not referenced in the case study)
- A series of evidence reviews and thematic evaluations on a new community health champion model
- A study commissioned by the Department of Health to produce and disseminate “research-based information to support better engagement with citizens to co-produce better health and well-being outcomes”.

The impact component of the case study describes the following activities and outputs:

- “Extensive public engagement and the production of outputs for practitioners and policy makers”
- An online searchable database about public involvement in public health with the results of 224 peer-reviewed publications
- Various endorsements, lay versions and policy briefings of the ‘People in Public Health’ review
- Contribution of the research team to various inquiries and consultations on lay health workers—for example to national reviews of obesity and exercise strategies and the Department of Health’s Third Sector Partnership Team.

Implicit in this case study, and more explicitly in a further paper published after it was submitted, is a collaborative programme of work characterised by the goals and standards of participatory research—such as power sharing, partnership synergy and trust. Also evident from the authors’ post-REF publications [35] is a distinct sense of non-linearity, with the different ‘academic’ and ‘engagement’ components of the study feeding bi-directionally into one another (e.g. when ‘lay’ informants supplied material that added to a systematic review) and the programme evolving in ways that were not initially anticipated (e.g. further NIHR funding to undertake a systematic review of peer-based interventions in prison settings).

## Box 1: Impact and the 2014 UK Research Excellence Framework (REF)

In the 2014 REF, 20 % of the score (and hence funding allocation) for each higher education institution was awarded for impact [1]. Impact was defined as occurring when academic research led to “… benefits to one or more areas of the economy, society, culture, public policy and services, health, production, environment, international development or quality of life, whether locally, regionally, nationally or internationally” (paragraph 62) and as “… manifested in a wide variety of ways including, but not limited to: the many types of beneficiary (individuals, organisations, communities, regions and other entities); impacts on products, processes, behaviours, policies, practices; and avoidance of harm or the waste of resources” (paragraph 63) [1].

RAND Europe advised the Higher Education Funding Council when developing the 2014 REF that the case study, written in narrative form and incorporating (where appropriate) both qualitative and quantitative data, was the format most likely to capture the complex links between research and impact [7]. Accordingly, each submitting unit (e.g. university department) provided an *impact template* describing infrastructure and activities oriented to maximizing impact and (depending on size) between two and forty-six 4-page *impact case studies*—examples of research programmes (1993–2013) and their impact beyond the higher education sector (2008–2013). In total, 6,975 impact case studies were submitted.

Panel A (Medicine) oversaw six sub-panels, including sub-panel A2 for community-based disciplines (Public Health, Health Services and Primary Care). Final impact scores across the Medicine sub-panels were high, with 95 % of 162 case studies in sub-panel A2, for example, scored as 4* (corresponding to ‘world leading’) or 3* (corresponding to ‘internationally excellent’) [32]. In an early calibration exercise, international assessors had given higher scores than UK assessors and encouraged a recalibration. As the final report from the Medicine Panel observed, almost every impact case study had drawn a compelling narrative linking research at the submitting institution with changes in policy, clinical or public health practice, service delivery, morbidity or mortality.

Impact scores from REF2014 were published in December 2014; all non-confidential case studies were placed in the public domain in January 2015. The final report from Main Panel A commented positively on the new component of impact (“Main Panel A were extremely impressed by the quality and breadth of research impact described. We believe that the collection of impact case studies from REF2014 provides a unique and powerful illustration of the outstanding contribution that research in the fields covered by this panel is making to health, wellbeing and society within and beyond the UK”) [32]. But the high overall scores, especially in comparison with impact scores in other panels, raised the question of whether impact had been fully and fairly assessed.
